# The Search for Cancer Biomarkers: Assessing the Distribution of INDEL Markers in Different Genetic Ancestries

**DOI:** 10.3390/cimb44050154

**Published:** 2022-05-19

**Authors:** Roberta B. Andrade, Giovanna C. Cavalcante, Marcos A. T. Amador, Fabiano Cordeiro Moreira, André S. Khayat, Paulo P. Assumpção, Ândrea Ribeiro-dos-Santos, Ney P. C. Santos, Sidney Santos

**Affiliations:** 1 Center of Oncology Research, Graduate Program in Oncology and Medical Sciences, Federal University of Pará, Belém 66073-005, Brazil; robertaborgesandrade@hotmail.com (R.B.A.); fabiano.ufpa@gmail.com (F.C.M.); khayatas@gmail.com (A.S.K.); assumpcaopp@gmail.com (P.P.A.); akelyufpa@gmail.com (Â.R.-d.-S.); npcsantos@yahoo.com.br (N.P.C.S.); 2 Laboratory of Human and Medical Genetics, Graduate Program in Genetics and Molecular Biology, Federal University of Pará, Belém 66075-110, Brazil; giovannaccavalcante@gmail.com (G.C.C.); marcosmata52@yahoo.com.br (M.A.T.A.)

**Keywords:** INDEL, cancer, genetic ancestry, Amazon, biomarkers

## Abstract

Cancer is a multifactorial group of diseases, being highly incident and one of the leading causes of death worldwide. In Brazil, there is a great variation in cancer incidence and impact among the different geographic regions, partly due to the genetic heterogeneity of the population in this country, composed mainly by European (EUR), Native American (NAM), African (AFR), and Asian (ASN) ancestries. Among different populations, genetic markers commonly present diverse allelic frequencies, but in admixed populations, such as the Brazilian population, data is still limited, which is an issue that might influence cancer incidence. Therefore, we analyzed the allelic and genotypic distribution of 12 INDEL polymorphisms of interest in populations from the five Brazilian geographic regions and in populations representing EUR, NAM, AFR, and ASN, as well as tissue expression in silico. Genotypes were obtained by multiplex PCR and the statistical analyses were done using R, while data of tissue expression for each marker was extracted from GTEx portal. We highlight that all analyzed markers presented statistical differences in at least one of the population comparisons, and that we found 39 tissues to be differentially expressed depending on the genotype. Here, we point out the differences in genotype distribution and gene expression of potential biomarkers for risk of cancer development and we reinforce the importance of this type of study in populations with different genetic backgrounds.

## 1. Introduction

Cancer is one of the leading causes of death worldwide [1], being considered a group of complex diseases that involve environmental, epigenetic, and genetic factors [2,3]. It is estimated that, in 2018, around 18 million new cases of cancer occurred in the world [1]. In Brazil, the National Cancer Institute (INCA) estimates that, for each year from 2020 to 2022, there were 625 thousand new cases, although there is a great variation in magnitude and in the cancer types among the different geographic regions of this country [4]. This occurs partly because Brazil has one of the most genetically heterogeneous populations in the world, composed mainly by Native American, European, and African contributions [5]. In addition, the biggest Japanese community outside Japan is in Brazil, estimated to be around 1.5 million people [6], which allows a certain degree of admixture between this population and the Brazilian population, mainly within the regions where this community is concentrated, North and Southeast of Brazil.

In the global literature, we may find several studies involving genetic markers related to cancer, mostly in case-control association studies, in which these are used to predict risk of development and/or prognosis of a certain type of cancer in different populations [7,8]. It is notable that, among different ethnic populations (also called continental populations), genetic markers commonly present diverse allelic frequencies [9]. However, in admixed populations, such as the Brazilian population, data on the distribution of this kind of markers are still limited.

In this work, we describe the allelic and genotypic distribution of 12 Insertion/Deletion (INDEL) polymorphisms, located in genes involved in important metabolic pathways associated with carcinogenesis, in populations from the five Brazilian geographic regions and in populations representing Europeans, Africans, Native Americans, and Asians. These genes and polymorphisms have been studied and associated with various types of cancer in different populations, such as bladder cancer [10], oral cancer [11], hepatocellular carcinoma [12], breast cancer [13,14,15], chronic lymphoblastic leukemia [16], colorectal cancer [17,18,19,20,21], thyroid cancer [22] and gastric cancer [23,24]. Thus, these markers were chosen based on the importance of each gene and their potential as an influence in tumor development.

The investigated markers were divided in three groups according to gene function: genomic stability and cell death (rs3834129, rs3730485, rs17878362, rs151264360, and rs3213239, respectively in *CASP8*, *MDM2*, *TP53*, *TYMS* and *XRCC1* genes); biometabolism and cell energy (rs8175347, rs28892005 and a 96 pb-insertion, respectively in *UGT1A1*, *CYP19A1* and *CYP2E1* genes); and immune response and inflammatory processes (rs3783553, rs79071878, rs28362491 and rs11267092, respectively in *IL1A*, *IL4*, *NFKB1* and *PAR1* genes).

## 2. Materials and Methods

This study included a population of 1411 non-related and cancer-free adult individuals, recruited in ten Brazilian states, in the years of 2009 and 2010, being 480 individuals from Pará (*n* = 360), Amazonas (*n* = 60) and Rondônia (*n* = 60) representing the North region; 370 individuals from Ceará (*n* = 135), Rio Grande do Norte (*n* = 175), Maranhão (*n* = 8) and Pernambuco (*n* = 52) representing the Northeast region; 186 individuals from Goiás (*n* = 101), Mato Grosso do Sul (*n* = 49) and Distrito Federal (*n* = 36) representing the Midwest region; 184 individuals from São Paulo representing the Southeast region; and 191 individuals from Rio Grande do Sul representing the South region. More details on the sampling approach may be found in previous studies [25,26].

In addition, we investigated a sample of 896 individuals representative of the main ethnic groups that contributed to the Brazilian population: 222 Native Americans (NAM) from nine tribes of the Brazilian Amazon (Tiriyó, Waiãpi, Zoé, Urubu-Kaapor, Awa-Guajá, Parakanã, Wai Wai, Gavião, Zoró) [27]; 211 Africans (AFR) from five different countries (Angola, Mozambique, Congo Republic, Cameroon, Ivory Coast) [28]; 270 Europeans (EUR) from two different countries (Portugal and Spain) [25,29]; and 193 Asians (ASN) from Japan [30]. By using a panel of ancestry informative markers (AIM), we have previously estimated the genomic ancestry of each group [30]. Informed consent for DNA analysis was obtained from all participants. Project approval was given by the Ethics Committee of Instituto de Ciências da Saúde, Universidade Federal do Pará.

### 2.1. DNA Extraction and Quantification

Samples of peripheral blood were collected from all individuals of the study and the DNA extraction was performed accordingly [31]. DNA quantification was performed with the NanoDrop 1000 spectrophotometer (Thermo Fisher Scientific, Wilmington, DE, USA).

### 2.2. Genotyping of Investigated Polymorphisms

Polymorphisms were genotyped by a single multiplex reaction with Master Mix QIAGEN^®^ Multiplex PCR kit (Qiagen, Hilden, Germany) and the primers are described in Table 1. PCR preparation protocol was done as described by Cavalcante et al. [32]. All polymorphisms are functional and correspond to INDEL of DNA fragments.

Multiplex PCR products were separated and analyzed by capillary electrophoresis on the ABI 3130 Genetic Analyzer instrument, using GS-500 LIZ as a pattern of molecular weight, G5 virtual filter matrix and POP7 (instrument and reagents by Thermo Fisher Scientific). Then, samples were analyzed with GeneMapper^®^3.7 software (also by Thermo Fisher Scientific).

### 2.3. Data Analyses

Allelic and genotypic frequencies were obtained by direct counting. Hardy-Weinberg Equilibrium (HWE) deviations were tested in Arlequin 3.1 software [33] and corrected by Bonferroni method. Differences in genotypic frequencies among Brazilian regions and parental populations were measured by chi-squared test (χ^2^ test, df = 2). FDR (False Discovery Rate) method was used to correct multiple analyses. All statistical analyses were performed in the statistical package R [34]. *p*-Value was considered significant if equal or lower than 0.05. In addition, to infer possible influences on cancer development, we assessed the Genotype-Tissue Expression (GTEx) Portal (https://gtexportal.org/home/, accessed on 1 May 2022) [35] to obtain the expression of each variant in different tissues.

## 3. Results

**Figure 1:**
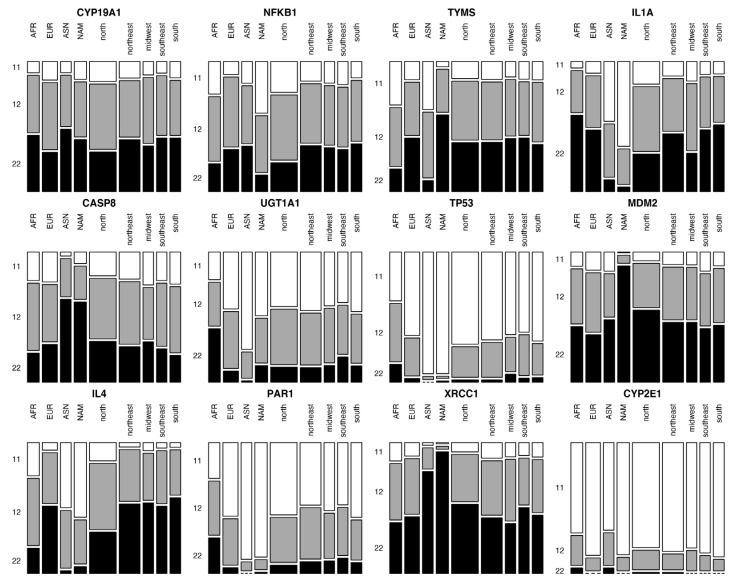
Genotype distribution of the markers across the studied populations. Genotype distribution of each marker in all analyzed populations: African, European, Asian, Native American and the five regions of Brazil (North, Northeast, Midwest, Southeast and South). 11, deletion/deletion; 12, deletion/insertion; 22, insertion/insertion.

The observed allele and genotype frequencies for the 12 markers investigated in the Brazilian population and the continental populations (AFR, NAM, EUR and ASN) are shown in Tables S1–S3; and the distribution of the genotypes is plotted in Figure 1.

When assessing HWE with correction for multiple testing for all markers in the different populations, we did not find any deviation from HWE in the admixed populations from Brazil. However, the markers in *CASP8*, *TP53* and *XRCC1* genes in Amerindian, *UGT1A1* gene in African, *NFKB1* gene in European, as well as *MDM2* and *IL4* genes in Asian populations, presented HWE deviation, indicating the distribution of these markers in such populations is not normalized according to HWE principles.

We then compared the genotypic distribution of the 12 markers among continental populations and the following results should be highlighted. Regarding biometabolism and cell energy markers (in *UGT1A1*, *CYP19A1* and *CYP2E1* genes), *CYP19A1* e U*GT1A1* did not present differences among populations in most comparisons, only in EUR vs. NAM, and *CYP2E1* also did not differ in the comparisons, except for AFR vs. ASN. As for genomic stability and cell death markers (*TYMS*, *XRCC1*, *CASP8*, *MDM2* and *TP53*), *XRCC1* and *CASP8* were significantly different in all populations, except for AFR vs. EUR; *TYMS* and *TP53* only presented statistical difference in AFR vs. ASN and NAM vs. ASN comparisons, respectively; and marker *MDM2* presented differences in the comparisons between NAM and all the other groups, but not in the other comparisons. Concerning markers of immune response and inflammatory processes (*IL1A*, *IL4*, *NKFB1* and *PAR1*), we observed significant differences for both *IL4* and *PAR1* in all comparisons; *IL1A* was different in all comparisons, but not in AFR vs. EUR; and *NFKB1* was only different in AFR vs. EUR and NAM vs. EUR. Due to the table size, *p*-values for these comparative analyses are shown in Table S4.

Moreover, we measured and analyzed δ (delta) values or mean frequencies among continental populations (Table 2). Among the investigated markers, the difference of δ values between NAM and AFR was 32%, between NAM and EUR was 23% and between EUR and AFR was 19%. In the comparisons involving ASN, an average delta value of 14% was estimated between ASN and NAM; 21% between ANS and EUR; and 26% between ASN and AFR.

In the comparison of geographic regions, the marker in IL4 was significantly different between North and the other populations of Brazil. Additionally, distribution of the marker in IL1A was significantly different between North and the regions South, Southeast and Northeast, and between Midwest and South. As for the polymorphism in NFKB1, it showed statistically significant difference between North and regions South and Southeast, but it was similar in all other comparisons.

Furthermore, in the GTEx analysis—performed to infer possible influences on cancer development, 39 tissues were found to be differentially expressed depending on the genotype in six of the 12 variants here investigated (Table 3). The variant that was differentially expressed in the highest number of tissues was rs3213239 (XRCC1), 30 tissues; followed by rs3834129 (CASP8), 15 tissues; rs3783553 (IL1A) and rs28362491 (NFKB1), six tissues each; rs151264360 (TYMS), three tissues; and rs11575899 (CYP19A1), two tissues.

Of all the found tissues, 12 seem to be regulated by more than one of the studied variants: cells—cultured fibroblasts (variants in *CASP8*, *NFKB1*, *XRCC1*), esophagus—mucosa (*CASP8*, *IL1A*, *XRCC1*), heart—atrial appendage (*NFKB1*, *XRCC1*), muscle—skeletal (*CASP8*, *NFKB1*, *XRCC1*), nerve—tibial (*CASP8*, *XRCC1*), pituitary gland (*CASP8*, *XRCC1*), skin—not sun exposed (NSE, suprapubic; *CASP8*, *CYP19A1*, *IL1A*, *NFKB1*), skin—sun exposed (SE, lower leg; *CASP8*, *CYP19A1*, *IL1A*), spleen (*CASP8*, *IL1A*), testis (*IL1A*, *NFKB1*, *TYMS*, *XRCC1*), thyroid (*CASP8*, *IL1A*, *XRCC1*) and whole blood (*NFKB1*, *XRCC1*).

## 4. Discussion

This study aimed to investigate and describe the frequencies of markers of interest (located in genes involved in important metabolic pathways associated with carcinogenesis) in populations from the five geographic populations of Brazil and in populations representing European, African, Native American, and Asian ancestries. These markers were divided according to gene functions.

In a previous study [5], the description of the group of markers of immune response and inflammatory processes was performed in the same populations investigated here, except for the Asian population. Regarding this group of markers, in addition to the results presented in that paper, it is possible to highlight that ASN population was different from all other continental populations for the investigated markers in *IL1A*, *IL4* and *PAR1*. As for the marker in *NFKB1*, it only differed between AFR and EUR and between NAM and EUR.

In the comparisons of geographic regions, marker *IL4* was significantly different between North and the other Brazilian populations. Besides that, our analysis also showed the distribution of the *IL1A* marker with statistical differences between North and the South, Southeast and Northeast regions, as well as between Midwest and South regions. The polymorphism in *NFKB1* was also significantly different between North and the regions South and Southeast. All other distributions of this group of markers were similar among these regions.

Regarding the investigated variants in genes of biometabolism and cell energy, not many studies can be currently found analyzing their distribution in populations of different genetic ancestries. However, a study by Fritsche et al. [36] compared genotypes of the 96-bp INDEL in *CYP2E1* gene in samples from individuals with African (African-American), European (European-American) and Asian (Taiwanese) genetic backgrounds and observed statistically significant differences between Europeans and Asians and between Europeans and Africans, but none between Asians and Africans, which corroborates our findings here. Concerning variant rs8175347 in *UGT1A1* gene, allele frequency of this variant has been reported as different when compared in groups of European, African, and Asian (including Japanese) ancestries [37]. No studies were found with the rs28892005 variant in *CYP19A1*.

As for variants in the group of genomic stability and cell death, there are some papers discussing their distribution in different populations in the global literature. For instance, a previous study by our research group compared the allele distribution of rs17878362 in *TP53* gene in populations of European, African, and Asian ancestry from 1000 genomes database [9], as well as a population from Northern Brazil, and observed statistical differences in all comparisons, with the exception of the one between Northern Brazil and European populations, which could be expected given the high contribution of European ancestry in this region [38]. However, it is notable that these frequencies significantly vary among different genetic backgrounds.

Similarly, the variant rs3730485 (also known as del1518) in *MDM2* gene has been investigated in different populations, particularly in connection with cancer development. For example, two independent studies involving different types of cancer in Chinese cohorts have reported a frequency of 30% of DEL allele in both groups of controls [12,39]. The study by Gansmo et al. [39] also investigated this variant in other populations, indicating the presence of the same allele in 38% and 42% in the African American and the Norwegian controls, respectively. Here, we found this allele in 33%, 38% and 33% of the African, European, and Asian groups, respectively, which seem to be close to the corresponding frequencies in these previous reports. To the best of our knowledge, this is the first study investigating this variant in NAM populations from the Brazilian Amazon.

Variant rs151264360 in *TYMS* gene has also been widely studied regarding cancer treatment in different regions. In this context, it has been associated with response to chemotherapy for colorectal cancer in a Mexican cohort, highlighting the importance of *TYMS* to cancer treatment in Latin American populations [40]. In that study, DEL allele was present in 33.0% of the participants, which was similar to the frequency of this allele in a study carried in a Slovak population (37.5%) [41]. A study by Summers et al. [42] reported a significant difference in the distribution of rs151264360 between African-Americans (DEL 53.75%) and Europeans (DEL 33.3%). These frequencies are like the ones observed here for African (DEL 58.0%) and European (DEL 36.0%) ancestries, which also showed significant differences.

Likewise, polymorphism rs3834129 in *CASP8* gene has been broadly studied regarding cancer, particularly cancer development. For instance, in a study by Pardini et al. [43], DEL allele of this variant was suggested as a protective effect to colorectal cancer in the multiple populations investigated, mostly from European countries. In these populations, the presence of DEL allele ranged from 45% to 52%. In addition, two independent studies investigating the distribution of this polymorphism in British cohorts in association to different diseases have reported the presence of DEL allele as 50% in the controls [44,45]. Similarly, a study by Chatterjee et al. [46] investigated the association of this marker with HPV infection and cervical cancer in South Africa and showed the presence of DEL allele in around 52% of the controls. Here, we found this allele in 50% and 47% of the African and European groups, respectively, which are also similar frequencies and corroborate these studies.

On the other hand, there are not many studies with the variant rs3213239 (*XRCC1* gene) in the specialized literature. Two studies by our research group have investigated this variant regarding cancer susceptibility in Northern Brazil, reporting association with acute lymphoblastic leukemia (ALL) [47], but not with gastric cancer or colorectal cancer [32]. Curiously, in the study by Carvalho et al. [47], not only the DEL/DEL genotype of this variant was associated with ALL, but also the genetic ancestry: NAM and EUR ancestries were associated with increased and decreased risk of developing ALL, respectively, highlighting the importance of investigating this variant in different populations.

Moreover, in the GTEx analysis, it is notable that the studied variants in *CASP8* and *XRCC1* appeared in most of the tissues showing more than one differentially expressed gene, nine each, of which six presented both markers: (i) cells—cultured fibroblasts, (ii) esophagus—mucosa, (iii) muscle—skeletal, (iv) nerve—tibial, (v) pituitary gland and (vi) thyroid. This suggests that these tissues are likely to be regulated by the variants in such genes, which are related to genomic stability and cell death.

Even though we did not find any works involving both *CASP8* and *XRCC1* and such tissues, there are a few studies in the global literature on the possible association of these genes and the development of different types of cancer, such as lung adenocarcinoma, breast cancer, gallbladder cancer, acute lymphoblastic leukemia, as well as gastric and colorectal cancers [32,47,48,49,50,51,52].

It is also notable that both skin tissues (SE and NSE) presented differential expression in *CASP8*, *CYP19A1* and *IL1A* and that NSE skin also presented this difference for *NFKB1*. This finding suggests a possible influence of this variant and gene on skin cancer development and reinforces previous studies that have reported the association of INS/INS genotype of this variant in *NFKB1* with an increased risk of developing melanoma in a Swedish and in a Brazilian population [53,54].

In addition to NSE skin, testis tissue also presented four differentially expressed variants (in *IL1A*, *NFKB1*, *TYMS* and *XRCC1* genes), the highest number of variants per tissue in this analysis. No studies were found about these specific variants in testis or these genes in testicular cancer, but the role of *IL1A*, *NFKB1* and *XRCC1* have been reported in Sertoli cells and other essential factors for spermatogenesis [55,56,57,58,59,60]. Hence, given their importance in testis function, these genes might also be involved in carcinogenesis in this tissue.

In summary, here we thoroughly analyzed the distribution of 12 polymorphisms in diverse populations (groups from European, African, Native American, and Asian populations, as well as groups from the five admixed Brazilian geographical regions) and tissue expression. All analyzed markers presented statistical differences in at least one of the population comparisons, and we found 39 tissues to be differentially expressed depending on the genotype, suggesting these markers might play a role in cancer distribution in different populations. Thus, we recommend future studies with larger cohorts to explore these novel observations, as this was the first study to investigate some of these markers in these populations. Based on our findings, we point out some potential biomarkers for risk of cancer development and we highlight the importance of this type of study in populations with different genetic backgrounds.

## Figures and Tables

**Table 1:** Technical characterization of the investigated polymorphisms.

Gene	ID	Size (bp)	Primers	Amplicon (bp)
*CASP8*	rs3834129	6	F-5′CTCTTCAATGCTTCCTTGAGGT3′ R-5′CTGCATGCCAGGAGCTAAGTAT3′	249–255
*CYP2E1*	-	96	F-5′TGTCCCAATACAGTCACCTCTTT3′ R-5′GGCTTTTATTTGTTTTGCATCTG3′	397–493
*CYP19A1*	rs28892005	3	F-5′TGCATGAGAAAGGCATCATATT3′ R-5′AAAAGGCACATTCATAGACAAAAA3′	122–125
*IL1A*	rs3783553	4	F-5′TGGTCCAAGTTGTGCTTATCC3′ R-5′ACAGTGGTCTCATGGTTGTCA3′	230–234
*IL4*	rs79071878	70 (1–3 repeats)	F-5′AGGGTCAGTCTGGCTACTGTGT3′ R-5′CAAATCTGTTCACCTCAACTGC3′	147/217/287
*MDM2*	rs3730485	40	F-5′GGAAGTTTCCTTTCTGGTAGGC3′ R-5′TTTGATGCGGTCTCATAAATTG3′	192–232
*NFKB1*	rs28362491	4	F-5′TATGGACCGCATGACTCTATCA3′ R-5′GGCTCTGGCATCCTAGCAG3′	366–370
*PAR1*	rs11267092	13	F-5′AAAACTGAACTTTGCCGGTGT3′ R-5′GGGCCTAGAAGTCCAAATGAG	265–277
*TP53*	rs17878362	16	F-5′GGGACTGACTTTCTGCTCTTGT3′ R-5′GGGACTGTAGATGGGTGAAAAG3′	135–141
*TYMS*	rs151264360	6	F-5′ATCCAAACCAGAATACAGCACA3′ R-5′CTCAAATCTGAGGGAGCTGAGT3′	148–164
*UGT1A1*	rs8175347	2 (5–8 repeats)	F-5′CTCTGAAAGTGAACTCCCTGCT3′ R-5′AGAGGTTCGCCCTCTCCTAT3′	133/135/137/139
*XRCC1*	rs3213239	4	F-5′GAACCAGAATCCAAAAGTGACC3′ R-5′AGGGGAAGAGAGAGAAGGAGAG3′	243–247

**Table 2:** Frequency of the shortest alleles of the 12 polymorphisms in the AFR, EUR, NAM and ASN populations, and the mean difference in frequency among populations (δ values).

	Frequencies				δ					
Markers	EUR	ASN	AFR	NAM	EUR/ASN	AFR/ASN	NAM/ASN	EUR/AFR	EUR/NAM	AFR/NAM
*IL1A*	0.3	0.69	0.22	0.82	0.39	0.47	0.13	0.08	0.52	0.6
*IL4*	0.26	0.69	0.58	0.77	0.43	0.11	0.08	0.32	0.51	0.19
*NFKB1*	0.38	0.40	0.52	0.64	0.02	0.12	0.24	0.14	0.26	0.12
*PAR1*	0.77	0.96	0.5	0.95	0.19	0.46	0.01	0.27	0.18	0.45
*UGT1A1*	0.68	0.89	0.45	0.70	0.21	0.44	0.19	0.23	0.02	0.25
*CYP19A1*	0.42	0.3	0.32	0.37	0.12	0.02	0.07	0.1	0.06	0.05
*CYP2E1*	0.94	0.8	0.83	0.95	0.14	0.03	0.15	0.11	0.01	0.12
*CASP8*	0.47	0.18	0.5	0.23	0.29	0.32	0.05	0.03	0.24	0.27
*TYMS*	0.36	0.65	0.58	0.21	0.29	0.07	0.44	0.22	0.15	0.37
*XRCC1*	0.33	0.1	0.83	0.02	0.23	0.73	0.08	0.5	0.31	0.81
*MDM2*	0.38	0.33	0.33	0.04	0.05	0.00	0.29	0.05	0.34	0.29
*TP53*	0.82	0.99	0.62	0.98	0.17	0.37	0.01	0.2	0.16	0.36
**Average**					0.21	0.26	0.14	0.19	0.23	0.32

**Table 3:** Differentially expressed variants in diverse tissues from GTEx. NES, Normalized Effect Size.

Gene	Variant ID	*p*-Value	NES	Tissue
*CASP8*	rs3834129	4.9 × 10^−13^	0.28	Cells-Cultured fibroblasts
		1.1 × 10^−9^	0.18	Esophagus-Mucosa
		1.5 × 10^−7^	−0.27	Pituitary gland
		2.6 × 10^−7^	−0.50	Brain-Cerebellum
		6.9 × 10^−7^	0.16	Adipose-Visceral (Omentum)
		7.3 × 10^−7^	−0.48	Brain-Frontal Cortex (BA9)
		0.0000019	−0.16	Thyroid
		0.0000030	0.18	Skin-Sun Exposed (Lower leg)
		0.0000035	0.22	Breast-Mammary Tissue
		0.000011	0.24	Spleen
		0.000024	−0.38	Brain-Cortex
		0.000055	0.14	Adipose-Subcutaneous
		0.00012	0.17	Skin-Not Sun Exposed (Suprapubic)
		0.00012	0.12	Muscle-Skeletal
		0.00023	−0.12	Nerve-Tibial
*CYP19A1*	rs28892005	1.1 × 10^−9^	0.20	Skin-Sun Exposed (Lower leg)
		0.00020	0.14	Skin-Not Sun Exposed (Suprapubic)
*IL1A*	rs3783553	3.9 × 10^−14^	0.35	Skin-Not Sun Exposed (Suprapubic)
		2.6 × 10^−11^	0.30	Skin-Sun Exposed (Lower leg)
		2.0 × 10^−7^	0.37	Spleen
		0.0000018	0.19	Testis
		0.000028	0.16	Esophagus-Mucosa
		0.00016	0.19	Thyroid
*NFKB1*	rs28362491	9.9 × 10^−14^	0.15	Muscle-Skeletal
		6.0 × 10^−8^	0.12	Cells-Cultured fibroblasts
		2.4 × 10^−7^	82	Whole Blood
		0.0000018	−0.12	Testis
		0.000099	−0.12	Heart-Atrial Appendage
		0.00021	−77	Skin-Not Sun Exposed (Suprapubic)
*TYMS*	rs151264360	6.6 × 10^−34^	0.66	Esophagus-Muscularis
		1.7 × 10^−22^	−0.28	Testis
		8.1 × 10^−17^	0.53	Esophagus-Gastroesophageal Junction
*XRCC1*	rs3213239	4.4 × 10^−45^	−0.43	Thyroid
		5.4 × 10^−42^	−0.53	Pancreas
		2.0 × 10^−26^	−0.49	Testis
		2.0 × 10^−24^	−0.58	Adrenal Gland
		3.8 × 10^−19^	−0.23	Muscle-Skeletal
		6.3 × 10^−15^	−0.32	Pituitary
		4.1 × 10^−13^	0.20	Nerve-Tibial
		4.2 × 10^−13^	−0.46	Brain-Hypothalamus
		8.7 × 10^−11^	−0.23	Colon-Sigmoid
		1.5 × 10^−10^	−0.54	Brain-Anterior cingulate cortex (BA24)
		4.8 × 10^−10^	−0.38	Brain-Caudate (basal ganglia)
		6.8 × 10^−10^	−0.23	Stomach
		1.1 × 10^−9^	−0.34	Ovary
		1.7 × 10^−9^	−0.24	Heart-Left Ventricle
		1.2 × 10^−8^	−0.41	Brain-Hippocampus
		2.0 × 10^−8^	−0.33	Brain-Nucleus accumbens (basal ganglia)
		3.7 × 10^−8^	−0.28	Liver
		3.8 × 10^−8^	−0.14	Colon-Transverse
		1.7 × 10^−7^	−0.29	Brain-Frontal Cortex (BA9)
		1.9 × 10^−7^	−0.29	Brain-Cortex
		2.0 × 10^−7^	−0.48	Brain-Amygdala
		4.5 × 10^−7^	−0.15	Esophagus-Mucosa
		0.0000022	−0.40	Minor Salivary Gland
		0.0000028	−0.55	Brain-Substantia nigra
		0.0000045	−0.30	Brain-Putamen (basal ganglia)
		0.000011	−84	Whole Blood
		0.000018	−78	Cells-Cultured fibroblasts
		0.000031	−0.24	Prostate
		0.000056	−0.15	Heart-Atrial Appendage
		0.00012	−94	Artery-Tibial

## Data Availability

The data presented in this study are available within the article or supplementary material.

## Supplementary Materials

The following supporting information can be downloaded at: https://www.mdpi.com/article/10.3390/cimb44050154/s1, Table S1. Allelic and genotypic frequencies of markers in Biometabolism and Cell Energy group. Table S2. Allelic and genotypic frequencies of markers in Genomic Stability and Cell Death group. Table S3. Allelic and genotypic frequencies of markers in Immune Response and Inflammatory Processes group. Table S4. *p*-Values of the comparative analyses of frequencies among all populations for each marker.
